# MiR-33a Controls hMSCS Osteoblast Commitment Modulating the Yap/Taz Expression Through EGFR Signaling Regulation

**DOI:** 10.3390/cells8121495

**Published:** 2019-11-22

**Authors:** Viviana Costa, Valeria Carina, Lavinia Raimondi, Angela De Luca, Daniele Bellavia, Alice Conigliaro, Francesca Salamanna, Riccardo Alessandro, Milena Fini, Gianluca Giavaresi

**Affiliations:** 1 IRCCS Istituto Ortopedico Rizzoli, 40136 Bologna, Italy; valeria.carina@ior.it (V.C.); lavinia.raimondi@ior.it (L.R.); angela.deluca@ior.it (A.D.L.); daniele.bellavia@ior.it (D.B.); francesca.salamanna@ior.it (F.S.); milena.fini@ior.it (M.F.); gianluca.giavaresi@ior.it (G.G.); 2 Department of Biomedicine, Neuroscience and Advanced Diagnostics, Section of Biology and Genetics, University of Palermo, 90133 Palermo, Italy; alice.conigliaro@unipa.it (A.C.); riccardo.alessandro@ior.it (R.A.); 3 Istituto per la Ricerca e L’Innovazione Biomedica, 90146 Palermo, Italy

**Keywords:** epithelial mesenchymal transition, mesenchymal stromal cells, microRNAs, regenerative medicine, EGFR signaling

## Abstract

Mesenchymal stromal cells (hMSCs) display a pleiotropic function in bone regeneration. The signaling involved in osteoblast commitment is still not completely understood, and that determines the failure of current therapies being used. In our recent studies, we identified two miRNAs as regulators of hMSCs osteoblast differentiation driving hypoxia signaling and cytoskeletal reorganization. Other signalings involved in this process are epithelial to mesenchymal transition (EMT) and epidermal growth factor receptor (EGFR) signalings through the regulation of Yes-associated protein (YAP)/PDZ-binding motif (TAZ) expression. In the current study, we investigated the role of miR-33a family as a (i) modulator of YAP/TAZ expression and (ii) a regulator of EGFR signaling during osteoblast commitments. Starting from the observation on hMSCs and primary osteoblast cell lines (Nh-Ost) in which EMT genes and miR-33a displayed a specific expression, we performed a gain and loss of function study with miR-33a-5p and 3p on hMSCs cells and Nh-Ost. After 24 h of transfections, we evaluated the modulation of EMT and osteoblast genes expression by qRT-PCR, Western blot, and Osteoimage assays. Through bioinformatic analysis, we identified YAP as the putative target of miR-33a-3p. Its role was investigated by gain and loss of function studies with miR-33a-3p on hMSCs; qRT-PCR and Western blot analyses were also carried out. Finally, the possible role of EGFR signaling in YAP/TAZ modulation by miR-33a-3p expression was evaluated. Human MSCs were treated with EGF-2 and EGFR inhibitor for different time points, and qRT-PCR and Western blot analyses were performed. The above-mentioned methods revealed a balance between miR-33a-5p and miR-33a-3p expression during hMSCs osteoblast differentiation. The human MSCs phenotype was maintained by miR-33a-5p, while the maintenance of the osteoblast phenotype in the Nh-Ost cell model was permitted by miR-33a-3p expression, which regulated YAP/TAZ through the modulation of EGFR signaling. The inhibition of EGFR blocked the effects of miR-33a-3p on YAP/TAZ modulation, favoring the maintenance of hMSCs in a committed phenotype. A new possible personalized therapeutic approach to bone regeneration was discussed, which might be mediated by customizing delivery of miR-33a in simultaneously targeting EGFR and YAP signaling with combined use of drugs.

## 1. Introduction

Bone homeostasis depends on the regulation of balance between tissue formation and resorption mediated by osteoblast and osteoclast activities, respectively. The imbalance between these two processes leads to several diseases, such as osteopenia, osteoporosis, etc. [1,2]. Bone regeneration is mediated by a well-orchestrated process that involves external and internal elements to promote formation of new mineralized tissues as receptor tyrosine kinase (RTK) signaling pathways, including epidermal growth factor (EGF), platelet-derived growth factor (PDGF), and fibroblast growth factor (FGF) [3]. Moreover, the cellular mechanisms underlying the impaired regeneration processes are not fully understood, making the improvements of current therapies less efficient [4,5].

MicroRNAs (miRNAs) are attractive candidates as multifunctional regulators of bone signaling [6] and have been investigated as new biomarkers of bone disease or regeneration in the last years. In our previous studies, we identified two miRNAs involved in bone regeneration: (i) miRNA-675-5p as a modulator of HIF-1α and Wnt/β-catenin signalings in human mesenchymal stromal cells (hMSCs) [6]; and (ii) miRNA-31-5p, a mechanosensitive miRNA involved in hMSCs hypoxia response [7]. Microarray analysis revealed that hMSCs and osteoblast cells showed strong differences in terms of miRNAs expression and relative signaling activation [8,9], such as epithelial-to-mesenchymal transition (EMT) pathway [9] and the epidermal growth factor (EGF) signaling [10], which are strongly correlated to hypoxia cell response during osteoblast differentiation [6].

EMT is a latent embryonic process that causes epithelial cells to lose their epithelial phenotype and to acquire a mesenchymal phenotype. It is regulated by the zinc-finger transcriptional factors: snail family transcriptional repressor 1 (SNAIL), snail family transcriptional repressor 2 (SLUG), and twist family basic helix-loop-helix (bHLH) transcription factor 1 (TWIST) [11]. SNAIL and SLUG are transcriptional repressors able to trigger the EMT process and regulate cell movement and adhesion [12]. SNAIL is known to regulate the bone homeostasis acting on RUNX2 and the vitamin D receptor (VDR) [13]. SLUG alone seems to negatively regulate adipogenesis signaling via a SNAIL-independent process [13,14]. In addition, the regulation on the Runx2 promoter is mediated by the direct interaction with the TWIST protein, which is a basic helix-loop-helix transcription factor involved in mesodermal and myoblast differentiation and in controlling osteoblastogenesis [15]. When TWIST is downregulated, Runx2 becomes functional and activates osteoblast formation by blocking adipocyte and chondrocyte differentiation [16].

A new hypothesis on osteoblast differentiation process was recently stated, involving two other proteins that act as mediators of EMT signaling: the transcription coactivator Yes-associated protein (YAP) and its paralog transcriptional coactivator with PDZ-binding motif (TAZ; also known as WWTR1), both interacting with the transcriptional enhancer factor TEF-1 TEAD/TEF, the first member of the TEAD family of transcription factors [13].

YAP, a key transcriptional factor downstream of the Hippo pathway and a mediator of EMT signaling, is expressed by committed osteoblast (OB) precursors or progenitors, matrix-producing OBs, lining cells, and matrix embedded osteocytes [17,18]. YAP is required to maintain cytoplasmic and nuclear pools of β-catenin, keeping the signaling activation during adult osteogenesis and bone homeostasis [19]. In addition, it was demonstrated that YAP improves osteogenesis differentiation of hMSCs by directly targeting SRY-Box Transcription Factor 2 (SOX2) and inhibiting adipogenesis [20]. TAZ was found to be induced by YAP expression [13] and was able to promote osteoblast differentiation of hMSCs by increasing Runt-related transcription factor 2 (Runx-2) expression and blocking the adipocytes differentiation [20].

Recent reports indicated that the crosstalk between epidermal growth factor receptor (EGFR) signaling and the Hippo pathway is involved in bone regeneration [21,22,23,24]. This was demonstrated when EGF affected bone and cartilage development, which influenced hMSCs proliferation and differentiation by the activation of the EGFR-ERK/MAPK signaling [25]. Tamama et al. [3] showed that the in vitro and the ex vivo EGF pretreatment did not affect the osteogenic differentiation of hMSCs, suggesting that it could be promising for their expansion.

On the contrary, it was demonstrated in several in vitro osteogenic systems such as MC3T3 cells, mouse bone marrow osteoblastic cells, and hMSCs that EGF-like ligands suppress osteoblast differentiation and inhibit the expression of bone markers at the transcriptional level in an EGFR-dependent manner. These data suggest that the activation of EGFR signaling in osteoprogenitors decreases the amounts of early bone markers and transcriptional factors and thus prevents these cells from entering into the differentiation process [26].

In this study, we wanted to understand the role of the miR-33a family during hMSCs osteoblast commitment and their relative targets using hMSCs as a model of stromal cells, hMSCs maintained in osteogenic medium as a model of pre-osteoblast cells, and Nh-Ost as a model of osteoblast cells [8,9]. Starting from the recent evidence that displayed a different miRNAs expression profiling between hMSCs and Nh-Ost as well as from our previous studies in which we demonstrated the roles of miR-675-5p and miR-31-5p in hypoxia and cytoskeletal modulation during hMSCs osteoblast differentiation [6,7], we decided to investigate another important signaling involved in hMSCs osteoblast commitment, such as EMT, for which it has been demonstrated that miR-33a shows a different mRNAs interaction.

Regarding the miR-33a family, it was demonstrated that high mobility group AT-hook 2 (HMGA-2) is a target of miR-33a-5p during osteoblast commitment, and that miR-33a is up-regulated in chemoresistant osteosarcoma and promotes osteosarcoma cell resistance to cisplatin by down-regulating TWIST (an EMT transcriptional factor) [27,28,29]. In addition, Price et al. demonstrated that miR-33a abolished mitochondrial function in different cell types [30], in accordance with our previous study in which we demonstrated that low intensity pulsed ultrasound stimulation induced the upregulation of mitochondrial function in the hMSCs cell model during osteoblast commitment. Through bioinformatic analysis, we aimed at fitting the scientific evidence obtained by us and by different authors, and we identified the miR-33a family (3p and 5p) as a possible modulator of YAP/TAZ during hMSCs osteoblast differentiation, thus driving the maintenance of stem and osteoblast phenotypes. Finally, the compensatory regulation of miR-33a expression during hMSCs commitment towards osteoblast differentiation through the modulation of YAP/TAZ complex expression—probably by acting on EGF signaling—was investigated.

## 2. Materials and Methods

### 2.1. Cell Culture and Reagents

Commercially available human mesenchymal stromal cells (hMSCs; Lonza, Walkersville, MD, USA) were cultured in Mesenchymal Stem Cell Growth Medium (MSCGM™; Bullet Kit, Lonza, Walkersville, MD, USA) in a humidified atmosphere of 5% of CO_2_ at 37 °C. To obtain osteogenic differentiation and consequent pre-osteoblast cells, hMSCs were treated with hMSC Mesenchymal Stem Cell Osteogenic Differentiation Medium (OM) (hMSC; Osteogenic Differentiation BulletKit™, Lonza). Commercially available normal human osteoblast cells (Nh-Ost; Lonza) were cultured in Osteoblast Growth Medium (OGM; Osteoblasts BulletKit™, Lonza). Culture medium was changed every three days, and cells were split at 70–80% of confluence using StemProAccutase (Gibco by Life Technologies Italia, Monza, Italy). Cells were used at an early passage for all experiments.

### 2.2. Cell Transfection

For cell transfection, Attractene Transfection Reagent (cat. number 1051531, Qiagen Srl, Milan, Italy) was used following the manufacturer’s indication. Briefly, hMSCs seeded at 150,000 cells/cm^2^ were transfected for 24 h with 15 pmol/mL hsa-miR-33a-5p mimic (4464066-MC12410, Life Technologies Italia), hsa-miR-33a-5p inhibitor (4464084-MH12410, Life Technologies Italia), hsa-miR-33a-3p mimic (4464066-MC12607, Life Technologies Italia), hsa-miR-33a-3p inhibitor (4464084- MH12607 Life Technologies Italia), or scrambled negative control (4464058, Life Technologies Italia). For all experimental groups, medium was collected at experimental times, and cells were processed for the following assays.

### 2.3. Epidermal Growth Factors (EGF) Cells Treatments

For EGF treatment, hMSCs were seeded at 150,000 cells/cm^2^ and treated with 50 µg of EGF (Recombinant Human EGF, 236-EG, R&D systems, Minneapolis, Canada) for 24, 48, 72, and 96 h to evaluate their viability and for 24, 36, and 48 h to perform mRNAs and proteins analyses.

### 2.4. Gefitinib Cells Treatments

To block EGFR action, hMSCs were seeded at 150,000 cells/cm^2^ and treated with 1µM of Gefitinib (Selleckchem, Houston, TX, USA) for 24, 36, and 48 h. After preliminary investigation (data not shown), we performed gene expression analyses after 36 h of treatments.

### 2.5. hMSC Viability (WST-1 Test)

WST-l colorimetric reagent (Roche Diagnostics GmbH, Manheim, Germany) was used to evaluate cell viability. Briefly, WST-1 reagent (10% *v*/*v*) was added to the cell monolayer in each well. After 24–36 and 48 h of EGF treatments, the formazan dye produced by viable cells was quantified spectrophotometrically at 450 nm by Bio-Rad Microplate Reader (Bio-Rad Laboratories, Hercules, CA, USA), and results were reported as percentage of viable cells compared to the untreated group.

### 2.6. RNA Extraction and Real-Time PCR

Total RNA was extracted using the commercially available IllustraRNAspin Mini Isolation Kit (GE Healthcare, Milan, Italy) according to the manufacturer’s instructions. RNA was reverse-transcribed to cDNA using the High Capacity cDNA Reverse Transcription Kit (Applied Biosystems, ThermoFisher Scientific, Rodano, Italy). Quantitative RT-PCR (qRT-PCR) analysis was performed in duplicates for each data point using custom-made primers (Invitrogen, Life Technologies Italia) (Table 1A–B). The mean threshold cycle was used for the calculation of relative expression using the Livak method against β-ACTIN as the reference gene [31]. For miRNA expression, 250 ng of RNA was reverse transcribed according to the manufacturer’s instructions (cat. number 4366596, TaqMan MicroRNA Reverse Transcription, Applied Biosystems Rodano, Italy, ThermoFisher Scientific, Italia). Taqman probes were used to analyze miR-33a-5p (4427975-ID002279, Applied Biosystem, ThermoFisher Scientific), miR-33a-3p (4427975–ID002113, Applied Biosystem, ThermoFisher Scientific), and U6 (4427975 Applied Biosystem, ThermoFisher Scientific). Changes in the target miRNA content were calculated in relation to the housekeeping U6 small nuclear 1.

### 2.7. Western Blot Analysis

SDS-PAGE and Western blot (WB) were performed according to standard protocols. Briefly, after respective transfection, hMCSs cells were lysed in lysis buffer containing 15 mM Tris/HCl pH7.5, 120 mM NaCl, 25 mM KCl, 1 mM EDTA, 0.5% Triton X100, and Halt Protease Inhibitor Single-Use cocktail (100×, Fisher Scientific Italia, Rodano, Italy). Whole lysates (15 µg per lane) were separated using 4–12% NovexBis-Tris SDS-acrylamide gels (Invitrogen, Life Technologies Italia), electro-transferred on nitrocellulose membranes (Bio-Rad Laboratories Srl, Segrate, Milan, Italy), and immunoblotted with the appropriate antibodies. Antibodies against the following proteins were used: SLUG [anti-rabbit Slug (C19G7) mAb-9585 Cell Signaling, Milan, Italy], YAP/TAZ [anti-rabbit YAP/TAZ (D24E4) Cell Signaling], α-Actin [monoclonal anti-α-actin (1A), sc32251, Santa Cruz Biotechnology, Heidelberg, Germany], EGFR (#2232; Santa Cruz Biotechnology), p44/42 MAPK(Erk1/2) (#9102; Cell Signaling). All secondary antibodies were obtained from Fisher Scientific Italia. Immunofluorescence was detected using a CCD high-resolution and high-sensitivity detection technology (ChemiDoc™ XRS+ System; Bio-Rad Laboratories Srl, Segrate, Milan, Italy).

### 2.8. OsteoImage^™^ Bone Mineralization Assay

In vitro mineralization of hMSC cells at 24 h for each type of transfections was assessed using the reagent from an OsteoImage^™^ Mineralization Assay kit (PA-1503-Lonza, Basel, Switzerland) to stain calcium deposits according to manufacturer’s instructions. The fluorescent signals from the HA portion were detected by a florescent plate reader, and the fluorescence was measured at 490–520nm wavelengths (GloMax^®^-Multi Detection System, Promega, Milan, Italy).

### 2.9. Statistical Analysis

Statistical analysis was performed by using R v.3.6.1 software (R Foundation for Statistical Computing Vienna, Austria, 2018; Available online: http://www.R-project.org/) [32]. Data are reported as mean ± SD at a significant level of *p* < 0.05. After having verified normal distribution (Shapiro–Wilk test) and homogeneity of variance (Levene test), Student *t* test was used to compare data.

## 3. Results

### 3.1. MiR-33a Family is Involved in the Maintenance of hMSCs and Osteoblast Phenotypes

**Figure 1:**
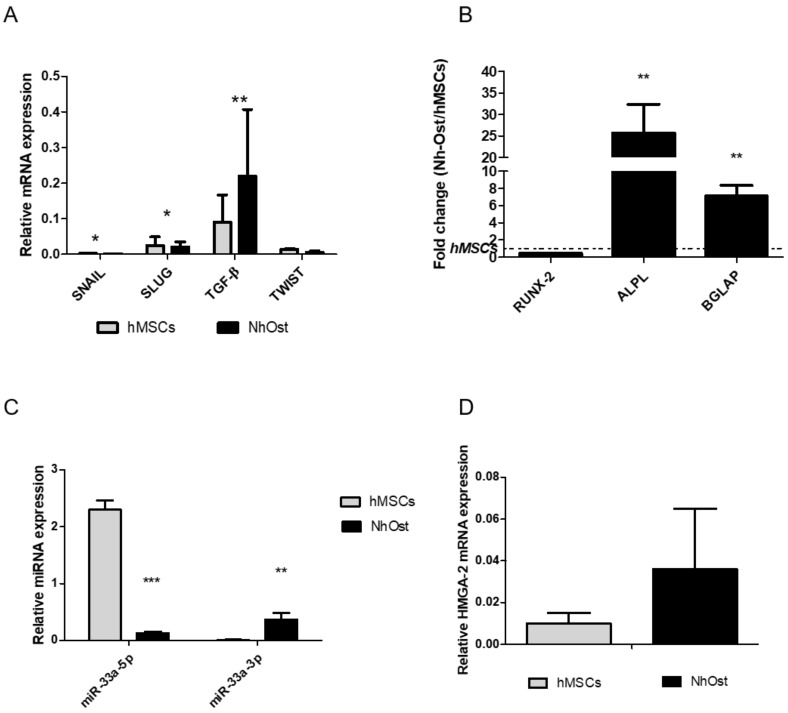
Investigation of human mesenchymal stromal cells (hMSCs) and normal human osteoblast cells (Nh-Ost) expression profiles cells by qRT-PCR analysis of the following genes: (**A**) epithelial to mesenchymal transition (EMT) markers: SNAIL, SLUG, TWIST, TGF-β; (**B**) osteoblast markers: RUNX-2, ALPL, BGLAP. MiR-33a-3p and 5p expression levels by qRT-PCR on both models (**C**) and relative mRNA expression levels of miR-33a-5p-target HMGA-2 (**D**) Quantitative RT-PCR data are expressed as relative mRNA or microRNAs (miRNAs) expression or fold of change (FOI) in gene expression (2^−ΔΔCt^) that occurred in Nh-Ost vs. hMSCs in each cell model. Student *t* test: * *p* < 0.05, ** *p* < 0.005, *** *p* <0.0005 between experimental group.

A gene expression analysis of the main EMT signaling molecules was carried out on hMSCs and Nh-Ost cells to highlight differences between them. As shown in Figure 1A, both cell lines displayed similar trend levels of EMT genes expression, even if in a statistically significant way when comparing them. However, a significant difference of osteoblast markers was observed (Figure 1B) comparing Alkaline phosphatase (ALP) (*p* = 0.002) and bone gamma-carboxyglutamic acid-containing protein (BGLAP) (*p* = 0.003) expression between Nh-Ost and hMSCs (Figure 1B). To investigate the possible involvement of specific miRNA on EMT signaling and osteoblast phenotype modulation, bioinformatic analysis of miRNA targets through TargetScan was performed, revealing that miR-33a targets different genes that could be involved in this signaling. To validate these bioinformatic data, the expression levels of miR-33a-3p and 5p were evaluated on hMSCs and Nh-Ost cells. As shown in Figure 1C, cell lines had a completely different expression of these miRNAs. hMSCs showed the highest expression of miR-33a-5p (*p* < 0.0005), while Nh-Ost showed high levels of miR-33a-3p expression (*p* < 0.0005). To confirm these differences, we investigated mRNA levels of miR33a-5p-target high mobility group AT-hook 2 (HMGA-2) in both cell types. Nh-Ost cells showed higher expression levels of HMGA-2 than hMSCs, in which it was not expressed in a significant manner (Figure 1D) [29,30].

### 3.2. MiR-33a Family Can Promote hMSCs Osteoblast Commitments

In order to better understand the role of miR-33a-5p in hMSCs commitment, we decided to perform gain and loss function studies on hMSCs cell model. We initially evaluated the effects of miR-33a-5p over-expression or inhibition by the transfection with specific mimic and antimiR or relative scrambles.

**Figure 2:**
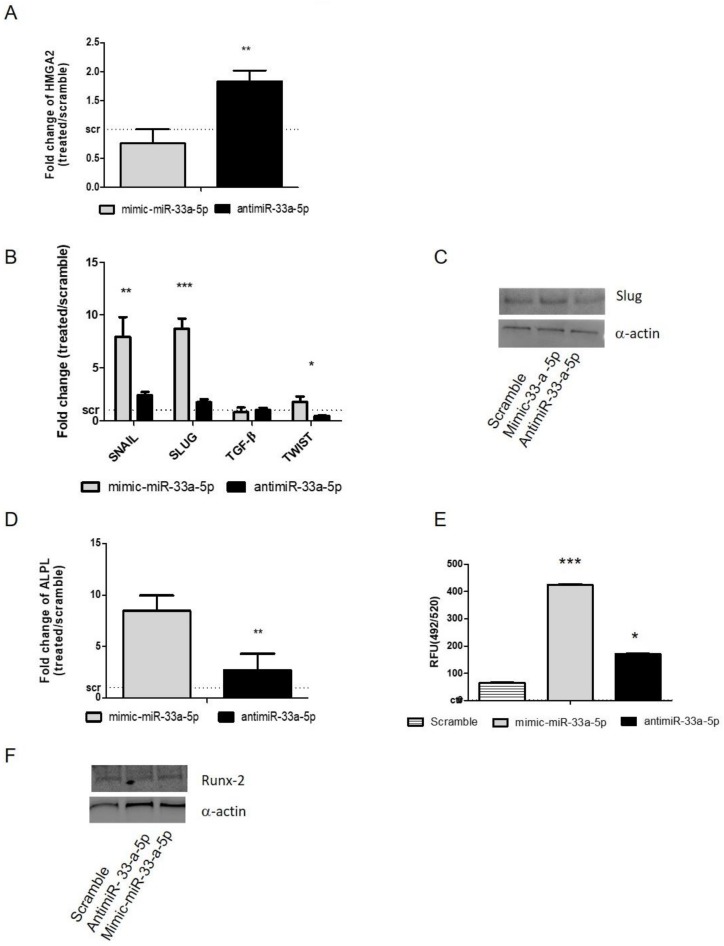
Study of miR-33a-5p-targets by gain and loss function assays; hMSCs after 24 h of transfection with specific mimic and antimiR were analyzed for the gene expression modulation by qRT-PCR and protein Western blot analysis. (**A**) miR-33a-5p targets HMGA-2; (**B**) EMT markers: SNAIL, SLUG, TWIST, TGF-β; (**C**) Slug; (**D**) ALPL. Quantitative RT-PCR data are expressed as fold of change (FOI) in gene expression (2^−ΔΔCt^) that occurred in mimic or antimiR vs. scramble groups. OsteoImage assay was performed to quantify the in vitro mineralization nodules, and data are shown as relative fluorescence units (RFU,492/520 nm) (**E**) Western blot analysis for Runx-2 (**F**) protein was performed on total cell extract after miR-33a-5p and 3p over-expression or inhibition. Student *t* test: * *p* < 0.05, ** *p* < 0.005, *** *p* < 0.0005 between experimental group.

After 24 h of mimic transfection, hMSCs showed downregulation of HMGA2 (*p* = 0.004), confirming it as an miR-33a-5p target (Figure 2A). Regarding the modulation of EMT molecules after over-expression or inhibition of miR-33a-5p on hMSCs, a strong increase of SNAIL (*p* = 0.008) and SLUG (*p* < 0.0005) after miR-33a-5p over-expression compared to antimiR-33a-5p transfection was observed; TWIST (*p* = 0.012) also showed a modulation after both transfections (Figure 2B). The effects on SLUG (*p* < 0.0005) mRNA were also confirmed by Western blot analysis (Figure 2C and Figure S1).

To understand the hypothetical role of miR-33a-5p on hMSCs osteoblast differentiation, we analyzed the expression levels of the alkaline phosphatase (ALPL) gene in our model after miRNA over-expression or inhibition. qRT-PCR data showed an increase of ALPL mRNA levels after miR-33a over-expression compared to miR-33a-5p antimiR transfection. (Figure 2D). These data were also confirmed by evaluation of mineralization nodules through OsteoImage assay (Figure 2E). Western blot analysis of Runx-2 protein expression (Figure 2E and Figure S2) showed a downregulation of the Runx-2 protein by antimiR-33a-5p transfection compared to scramble and mimic groups.

**Figure 3:**
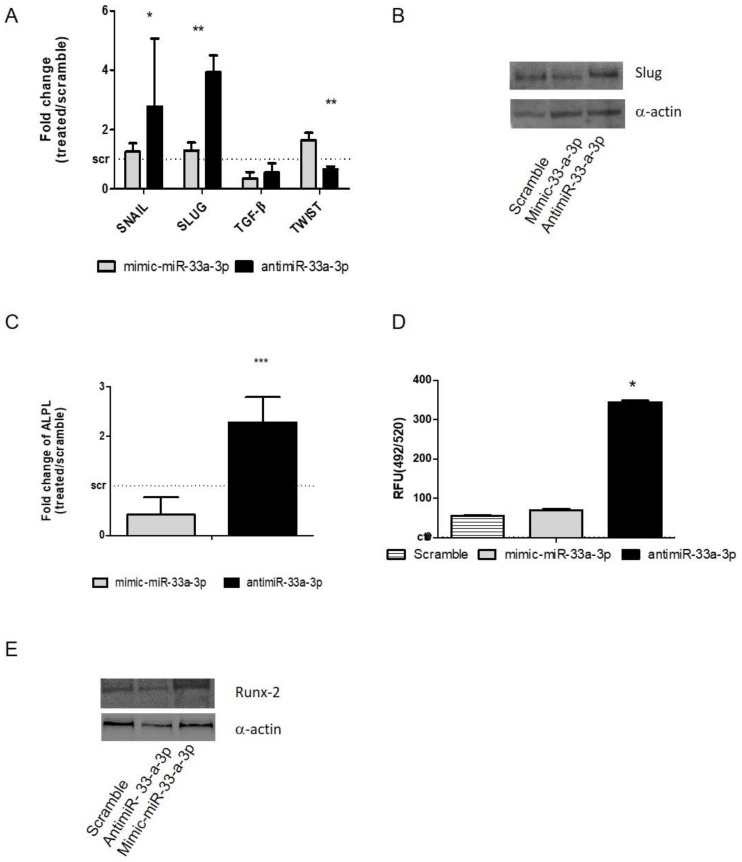
Study of miR-33a-3p-targets by gain and loss of function assays; hMSCs after 24 h of transfection with specific mimic and antimiR were analyzed for the gene expression modulation by qRT-PCR and protein Western blot analysis. (**A**) EMT markers: SNAIL, SLUG, TWIST, TGF-β; (**B**) Slug; (**C**) ALPL. OsteoImage assay was performed to quantify the quantitative in vitro mineralization nodules, and data are shown as RFU (492/520 nm) (**D**) Western blot analysis for Runx-2 (**E**) protein was performed on total cell extract after miR-33a-5p and 3p over-expression or inhibition. Quantitative RT-PCR data are expressed as fold of change (FOI) in gene expression (2^−ΔΔCt^) that occurred in mimic or antimiR vs. scramble groups. Student *t* test: * *p* < 0.05, ** *p* < 0.005, *** *p* < 0.0005 between experimental group.

As for miR-33a-5p, gain and loss of function assays were performed with miR-33a-3p on hMSCs (Figure S3). Surprisingly, a significant upregulation of SNAIL (*p =* 0.032) and SLUG (*p* = 0.002) mRNAs was observed through qRT-PCR analysis on hMSCs after antimiR-33a-3p transfection, while no modulation of TGF-β was observed (Figure 3A). Western blot analysis of the SLUG protein demonstrated that miR-33a-3p inhibition or over-expression induced the same effects on hMSCs compared to the scramble group, suggesting that miR-33a-3p is involved only in SLUG mRNA expression (Figure 3B and Figure S4). The TWIST gene showed a significant increase after mimic-miR-33a-3p transfection (*p* = 0.003).

Furthermore, the effects of miR-33a-3p over-expression or inhibition on osteoblast differentiation ability were investigated. hMSCs showed an important increase of ALP mRNA (*p* = 0.001), as confirmed by the evaluation of mineralization nodules (Figure 3C–D), while Runx-2 protein investigation after antimiR-33a-3p transfection showed little increase compared to mimic-miR-33a-3p transfection (Figure 3E and Figure S5), suggesting probable action of miR-33a-3p to ALPL expression independently by the modulation of its transcriptional factor Runx-2.

### 3.3. Modulation of miR-33a-5p and miR-33a-3p Expression Regulates the Activation of EMT Signaling in Pre-Osteoblast Cells

**Figure 4:**
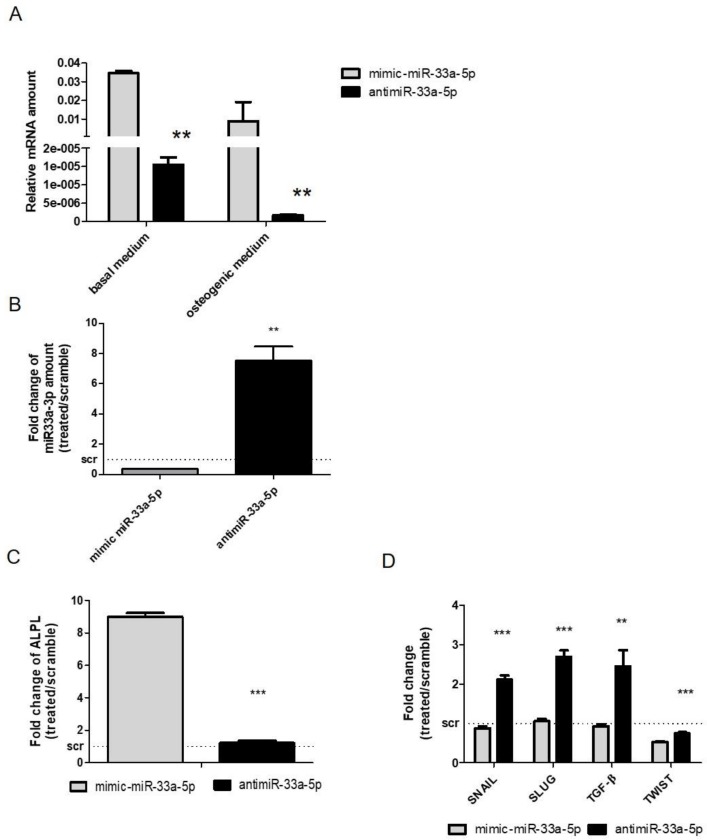
Evaluation of miR-33a-5p over-expression or inhibition on hMSCs maintained in Osteogenic Differentiation Medium (OM) for 3 days. qRT-PCR analysis was performed for: (**A**) miR-33a-5p; (**B**) miR-33a-3p; (**C**) ALPL; (**D**) SNAIL, SLUG, TWIST, TGF-β. Quantitative RT-PCR data are expressed as relative miRNAs amount or as fold of change (FOI) in gene expression (2^−ΔΔCt^) that occurred in mimic or antimiR vs. scramble groups. Student *t* test: * *p* < 0.05, ** *p* < 0.005, *** *p* < 0.0005 between experimental group.

After the investigation on the effects of miR-33a-5p and 3p over-expression or inhibition on hMSCs, a comparison was made between the miR-33a-5p expression levels on hMSCs maintained in OGM medium and hMSCs in basal medium. Figure 4A shows a modulation of miR-33a family expression between the cell groups, confirming a role of miR-33a-5p on hMSCs osteoblast commitments.

To evaluate the contribution of miR-33a-5p on osteoblast commitment, we performed gain and loss of function studies. qRT-PCR analysis of miR-33a-5p and miR-33a-3p expression after mimic and antimiR-33a-5p transfection, respectively (Figure S6), showed a particular decrease of miR-33a-3p expression after mR-33a-5p over-expression (*p* = 0.001), while an increase of miR-33a-3p expression after miR-33a-5p depletion was observed (*p* = 0.006) (Figure 4B). Gain and loss of function studies revealed that the over-expression of miR-33a-5p induced the downregulation of HMGA2 miR-target (*p* < 0.0005), confirming its action on a specific target (Figure S7).

To evaluate the osteoblast phenotype of hMSCs after maintenance in OM, mRNA level of ALP was evaluated, where an upregulation of ALP after mimic transfection was observed compared to antimiR transfection (*p* < 0.0005) (Figure 4C).

Regarding the modulation of EMT signaling after miR-33a-5p over-expression or inhibition, a significant increase in SNAIL (*p* < 0.0005) and SLUG (*p* < 0.0005) mRNAs was found after antimiR-33a-5p transfection in the same manner as that obtained after miR-33a-3p inhibition (Figure 4D). For the first time, an increase in TGF-β mRNAs was observed after miR-33a-5p inhibition compared to its over-expression (*p* = 0.003), and a significant decrease of TWIST mRNA was found after both transfections (*p* < 0.0005) (Figure 4D).

### 3.4. YAP/TAZ: Possible Mediators of miR-33a Effects During hMSCs Osteoblast Differentiation Process

**Figure 5:**
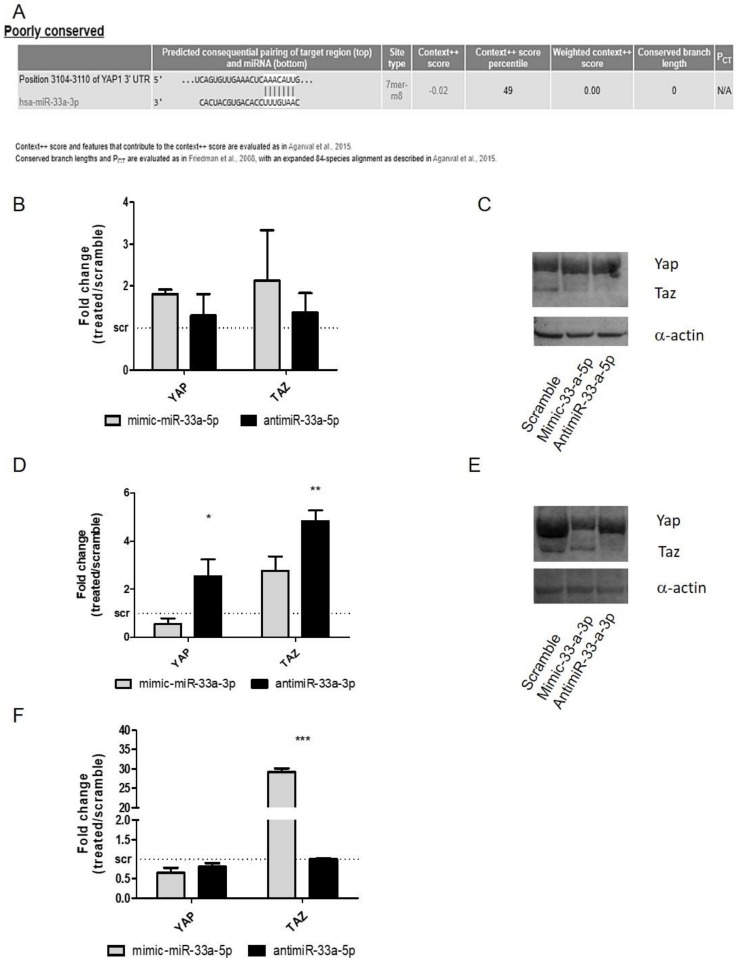
Analysis of Yes-associated protein (YAP) and PDZ-binding motif (TAZ) gene expression on hMSCs after gain and loss function studies. Bioinformatic analysis through TargetScanPrediction Software on miR-33a predicted target. (**A**) Predicted sequences of YAP. qRT-PCR was performed after 24 h of specific transfection. YAP and TAZ expression levels were evaluated on hMSCs after miR-33a-5p over-expression or inhibition (**B**) or after miR-33a-3p over-expression or inhibition (**D**) or after miR-33a-5p over-expression or inhibition on hMSCs maintained in OB (**F**) Western blot analysis for YAP/TAZ (**C**–**D**) proteins was performed on total cell extract after miR-33a-5p and 3p over-expression or inhibition. Quantitative RT-PCR data are expressed as fold of change (FOI) in gene expression (2^−ΔΔCt^) that occurred in mimic or antimiR vs. scramble groups. Student *t* test: * *p* < 0.05, ** *p* < 0.005, *** *p* < 0.0005 between experimental group.

To investigate the possible target of miR-33a on EMT signaling modulation during hMSCs osteoblast differentiation, we performed a bioinformatic analysis on miR-33a predicted EMT-targets. YAP was identified as a predicted target of miR-33a-3p, as shown in Figure 5A. To confirm our hypothesis, YAP and TAZ mRNA expression levels were analyzed on hMSCs transfected with mimic-miR-33a-3p or 5p and antimiR-33a-3p or 5p.

No modulation was found after the over-expression of miR-33a-5p or its depletion for YAP/TAZ mRNAs (Figure 5B). Western blot analysis of YAP and TAZ (Figure 5C and Figure S8) showed an increase of YAP/TAZ proteins only after mimic-miR-33a-5p transfection, suggesting probable action of miR on the specific regulator of their protein expression rather than directly to mRNAs. It should be noted that YAP (*p* = 0.009) and TAZ (*p* = 0.008) mRNAs showed a significant increase after antimiR-33a-3p transfection compared to mimic transfection (Figure 5D), as confirmed by Western blot analysis of YAP and TAZ proteins (Figure 5E and Figure S9).

Regarding hMSCs maintained in osteogenic medium, YAP and TAZ mRNAs modulation analysis were performed. YAP did not show any variation, while TAZ presented an increase after mimic-33a-5p over-expression compared to inhibition (*p* < 0.0005) (Figure 5F).

### 3.5. EGF Treatment Induce the Expression of miR-33a-3p and YAP/TAZ Modulation on hMSCs

In order to better understand the role of the miR-33a family on hMSCs and Nh-Ost phenotypes, we evaluated the effects of EGF treatment on hMSCs. The first time, we performed a WST1 assay on hMSCs treated with EGF, and no changes on hMSCS viability were observed during the different time points (Figure S10).

**Figure 6:**
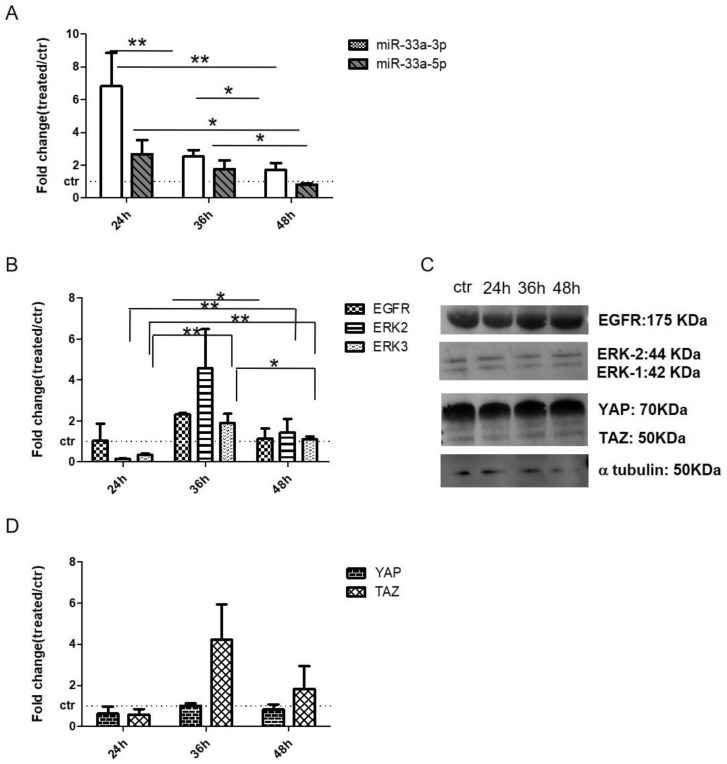
Analysis of miRNAs and gene expression on hMSCs treated with EGF. qRT-PCR was performed after 24 h, 36 h, and 48 h of treatments: (**A**) miR-33a-3p and miR-33a-5p; (**B**) EGFR, ERK-2, and ERK-3; (**D**) YAP and TAZ. Western blot analyses for EGFR, ERK-2, ERK-1, YAP, TAZ and α-tubulin (**C**) proteins were performed on total cell extract after EGF treatments. Quantitative RT-PCR data are expressed as fold of change (FOI) in gene expression (2^−ΔΔCt^) that occurred in hMSCs after treatments vs. control groups. Student *t* test: * *p* < 0.05, ** *p* < 0.005, *** *p* < 0.0005 between experimental group.

qRT-PCR analysis revealed that hMSCs displayed an increase of miR-33a-3p levels after 24 h of treatment and strong downregulation after EGF stimulation at 36 h; on the contrary, miR-33a-5p showed no changes during the experimental times (Figure 6A). Regarding the modulation of EGF signaling, hMSCs showed an increase in EGFR, extracellular signal-regulated kinases-2 (ERK2), and extracellular signal-regulated kinases-3 (ERK3) mRNAs at 36 h and proteins at 48 h of EGF treatment compared to other time points (Figure 6B,C and Figure S11).

To evaluate the crosstalk between EGF and Hippo signaling, we analyzed the modulation of YAP and TAZ after EGF treatment. qRT-PCR and Western blot analysis revealed that hMSCs displayed an increase in the expression of mRNAs TAZ and YAP after 36 h of treatment, while YAP and TAZ proteins showed an increase of expression after 48 h of treatments (Figure 6C,D, and Figure S12).

All these data suggest a different time of action of EGF signaling on mRNA and relative protein expression [33,34].

**Figure 7:**
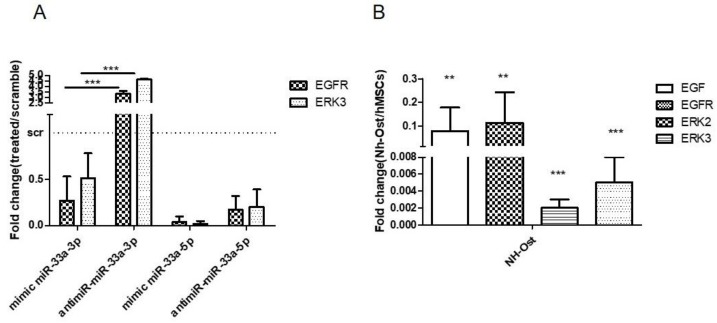
Analysis of gene expression on hMSCs after EGF treatments and EGFR signaling gene expression investigation on Nh-Ost. qRT-PCR was performed after 24 h of specific transfection on hMSCs. EGFR and ERK3 expression levels were evaluated on hMSCs after miR-33a-3p over-expression or inhibition or after miR-33a-5p over-expression or inhibition (**A**) qRT-PCR was performed on Nh-Ost, EGF, EGFR, ERK-2, and ERK-3 (**B**) Quantitative RT-PCR data are expressed as fold of change (FOI) in gene expression (2^−ΔΔCt^) that occurred in hMSCs after treatments vs. scramble groups and in Nh-Ost vs. hMSCs cells. Student *t* test: * *p* < 0.05, ** *p* < 0.005, *** *p* < 0.0005 between experimental group.

Regarding the modulation of miR-33a family expression after EGF treatments, we investigated by gain and loss of function studies whether or not miR-33a-3p regulated EGF signaling on the hMSCs model. qRT-PCR analysis highlighted that hMSCs showed strong downregulation of EGFR genes after mimic miR-33a-3p transfection, while upregulation of EGFR (*p* < 0.0005), ERK2, and ERK3 (*p* = 0.0008) mRNAs was observed after transfection with the miR-33a-3p inhibitor. No changes were observed after miR-33a-5p mimic and inhibitor transfection (Figure 7A). To support the data about the involvement of EGF on osteoblast phenotype, we investigated the modulation of EGF signaling on the Nh-Ost model. qRT-PCR analysis revealed that cells did not express EGF (*p* = 0.0019), EGFR (*p* = 0.0035), ERK2 (*p* < 0.0005), and ERK3 (*p* < 0.0005) mRNAs (Figure 7B). These data confirm that EGF is downregulated in osteoblast cells and during hMSCs osteoblast commitment.

**Figure 8:**
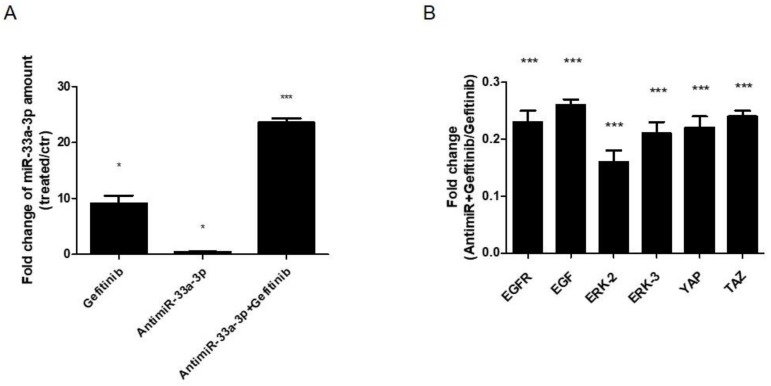
Analysis of gene expression on hMSCs after Gefitinib treatments (36 h) and antimiR-33a-3p transfection (24 h). qRT-PCR was performed after 36 h of treatments: (**A**) miR-33a-3p; (**B**) EGFR-2, EGF, ERK-2, ERK3, YAP, and TAZ. Quantitative RT-PCR data are expressed as fold of change (FOI) in gene expression (2^−ΔΔCt^) that occurred in hMSCs after treatments vs. scramble groups and hMSCs after antimiR-33a-3p transfection and Gefitinib treatments vs. Gefitnib groups. Student *t* test: * *p* < 0.05, ** *p* < 0.005, *** *p* < 0.0005 between experimental group.

Finally, to investigate the involvement of miR-33a-3p on EGF signaling modulation, we treated hMSCs for 24, 36, and 48 h with 10 μM di Gefitinib. qRT-PCR analysis showed that cells showed an increase of miR-33a-3p expression compared to control cells after 36 h of Gefitinib treatment (Figure 8A). At the same time point, the cells showed strong downregulation of YAP/TAZ, EGFR, and ERK2-3 mRNAs after antimiR-33a-3p transfection and Gefitinib co-treatment compared to Gefitinib treated cells (Figure 8B). These data confirmed a possible regulation between miR-33a-3p and EGFR signaling into YAP/TAZ expression.

## 4. Discussion

Despite various in vitro and in vivo studies, the molecular mechanisms of the lineage commitment of hMSCs to osteoblast are not yet fully understood. Recent evidence suggested a role of some signaling in hMSCs osteoblast differentiation processes, such as hypoxia, EMT, and EGFR signaling [10,26,35,36].

In our previous studies, a correlation between hypoxia activation [37] during osteoblast differentiation and miR-675-5p expression was identified in vitro on hMSCs. Interestingly, a key role of miR-675-5p in hypoxia [37] and EMT cross-talk was observed in colon cancer cell lines [38]. The role of hypoxia as an inductor of EMT signaling activation is well known in tumor progression [38]. In this study, the role of the EMT pathway during osteoblast hMSCs commitments was investigated for the first time.

Thus, the different expression patterns of EMT markers by qRT-PCR on hMSCs and Nh-Ost cells were investigated; no significant modulation on EMT signaling was observed in either cell line, despite a different osteoblast marker expression highlighted between cell models. Through bioinformatic analysis, the possible involvement of miR-33a in EMT control in the maintenance of hMSCs and Nh-Ost phenotypes was thoroughly investigated.

MiR-33 is a miRNA family that is highly conserved from Drosophila to humans [39]. Two isoforms of miR-33, miR-33a and miR-33b, are expressed in humans. However, only one miR-33 isoform, miR-33a, is expressed in mice and conserved in humans. Human miR-33a has two subtypes, miR-33a-3p and miR-33a-5p, which correspond to miR-33-3p and miR-33-5p in mice, respectively. A recent study demonstrated that miR-33a-5p might be used as a delivery system to the treatment of pathological osteopenia derived by mechanical unloading [40], as was shown by the submission of miR-103a. [29]. However, Wang et al. investigated the osteopenia mouse model and showed that the in vivo over-expression of miR-33a-5p was able to induce the promotion of osteoblasts activity and a relative increase of osteoid formation [29], overcoming the disadvantages shown by a single injection of miR103a, such as side effects in other tissues and rapid biodegradation [29].

MiR-33a provides some important functions, such as the regulation of cellular mechanotransduction sensing, mitochondrial biogenesis, and mitochondrial fatty acid oxidation [30,41]. It is known that, in bone tissue, miR-33a plays a role in osteosarcoma chemoresistance by down-regulating TWIST [42] and in osteoblast differentiation after mechanical stimulations [43,44]. Moreover, it was demonstrated that miR-33a-5p regulates osteoblast differentiation in MC3T3-E1 cells, and its over-expression increases the expression of *Runx2*, *Osterix*, and *Alp*, indicating that miR-33-5p promotes osteoblast differentiation in vitro [27,43].

In our in vitro models, we demonstrated a different expression of two miR-33a; hMSCs displayed a strong expression of miR-33a-5p, while Nh-Ost cells had a significant expression of miR-33a-3p, as confirmed by the analysis of the miR-33a-5p-target HMGA (Figure 1B–C) [28]. These data suggest a pivotal role of the miR-33a family in driving and maintaining the osteoblast phenotype, which probably drive EMT signaling activation.

To investigate the role of the miR-33a family in hMSCs commitment, we performed gain and loss of function studies. Gene expression profiling and protein analysis revealed that miR-33a-5p over-expression was able to modulate EMT signaling activation by up-regulating SNAIL and SLUG mRNAs and the SLUG protein at the same time (Figure 2B). In addition, the over-expression of miR-33a-5p promoted the acquisition of the osteoblast phenotype, as shown by ALP mRNA expression (Figure 2C) and the quantification of mineralization nodules formation (Figure 2D).

Unexpectedly, the antimiR-33a-3p transfection was able to promote the increase of the same gene that was modulated by miR-33a-5p over-expression as SNAIL and SLUG (Figure 3A).

An interesting point in question was to determine the role of the miR-33a family in pre-osteoblast cells in order to identify a timeline of miR-33a-3p and miR-33a-5p expression during hMSCs osteoblast commitment. For this reason, we obtained pre-osteoblast cells by maintaining hMSCs in osteoblast medium [45], and a significant difference on miR-33a-3p and 5p expression patterns was evidenced (Figure 4A). Surprisingly, the inhibition of miR-33a-5p on hMSCs maintenance in osteogenic medium induced an increase of miR-33a-3p expression, suggesting possible activation of a cell control mechanism from hMSCs to maintain a balance of miRNAs expression during the osteoblast differentiation process (Figure 4B). As expected, we obtained a positive regulation of EMT signaling and a consequent the increase of osteoblast marker ALP (Figure 4C).

To understand the role of the miR-33a family on EMT signaling modulation during hMSCs osteoblast differentiation, we focused our attention on the identification of a new possible target of miR-33a family. Through TargetScanPrediction analysis, we identified YAP as a predicted target of miR-33a-3p (Figure 5A). The role of YAP/TAZ on EMT signaling modulation in tumor progression has already been established and the synergic role of YAP/TAZ on bone regeneration was demonstrated in an in vivo model of Yap-CKO mice. It was showed a compensatory effect of TAZ in terms of numbers of osteoblast (OBs) derived by hMSCs, reducing only 50% the OB^+^ cells during growth [13]. In addition, it was reported that YAP and TAZ are associated with SMADs to promote transcription of transforming growth factor-β (TGF-β) and bone morphogenetic protein (BMP) target genes and functions, allowing the activation of EMT signaling [13,14]. In this light, recent reports have established the compensatory roles for YAP/TAZ, as well as SNAIL and SLUG [20]. SNAIL and SLUG control the transcriptional and post-transcriptional regulation of YAP/TAZ in mouse and human MSCs, and they were identified in the promoter regions of some YAP/TAZ-TEAD-targeted genes, such as RUNX-2 [14,44].

In this study, we investigated the modulation of both proteins after gain and loss of function studies. MiR-33a-5p over-expression or inhibition did not modulate YAP/TAZ expression on hMSCs (Figure 5B), while the antimiR-33a-3p transfection was able to induce a strong increase of YAP and TAZ in terms of mRNAs and proteins (Figure 5D). Regarding, the gain and loss of function studies on pre-osteoblast cells (hMSCs maintained in osteogenic medium), an increase of TAZ mRNA expression was observed alone after mimic-mR-33a-5p transfection (Figure 5E). The data obtained suggested that when hMSCs acquired a pre-osteoblast phenotype, miR-33a-5p can improve the expression of TAZ mRNA probably by blocking a specific TAZ inhibitor factor and consequently inducing the increase of TAZ expression.

In order to understand the double role of miR-33a-3p and 5p on the osteoblast commitment of hMSCs, we investigated EGFR signaling involved in this process [26]. hMSCs showed the increase of miR-33a-3p compared to miR-33a-5p when treated with EGF (Figure 6A). At the same time, we observed an inverse regulation of EGFR signaling molecules; YAP and TAZ were up- and downregulated when hMSCs expressed miR-33a-5p and miR-33a-3p, respectively (Figure 6B-C). These data confirmed the role of EGFR signaling during osteoblast commitments; in fact, EGFR signaling was up-regulated in hMSCs that downregulated miR-33a-3p and was downregulated after mimic-miR-33a transfection (Figure 7A). These data also confirmed that the Nh-Ost model physiologically expressed miR-33a-3p and consequently downregulated the expression of EGFR signaling (Figure 7B). In this sight, many studies showed that EGFR signaling is involved in the maintenance of hMSCs pluripotent cells. In fact, current results showed that EGF-treated hMSCs down-regulated osteoblast markers compared to control cells over time points (data not shown).

**Figure 9:**
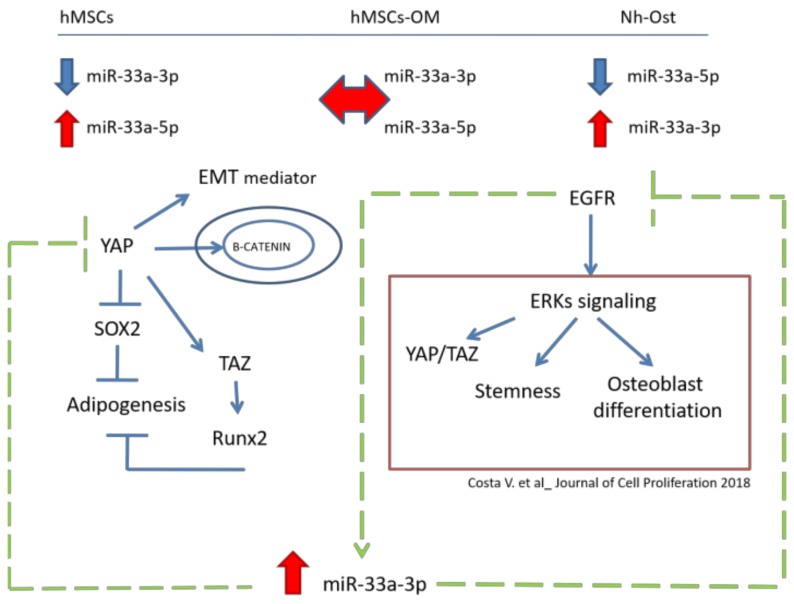
MiR-33a molecular mechanisms during hMSCs osteoblast commitment. In the upper part of the figure are the reported miR-33a expression levels in hMSCs, pre-osteoblast (hMSCs OM), and Nh-Ost models. The central part of the figure shows the molecular mechanism in which YAP and ERKs are involved. The green arrows report new evidence about the role of miR-33a-3p in the regulation of EGFR and YAP/TAZ signaling.

To highlight the role of miR-33a-3p on EGFR regulation, hMSCs were treated with an EGFR inhibitor (Gefitinib) in the presence or the absence of the inhibitor, miR-33a-3p. Gefitinib is a potent and selective inhibitor of EGFR tyrosine kinase (EGFRTK) normally used in tumor therapy. Gefitinib specifically inhibits cell proliferation in vitro, and it also exhibits a broad anti-tumor spectrum against non-small cell lung cancer (NSCLC) as well as prostate, colorectal, and ovarian cancers in vivo. In vitro studies demonstrated that Gefitinib also inhibits vascular endothelial growth factor (VEGF) production in tumor cells, leading to suppression of angiogenesis [46]. The presence of Gefitinib was able to induce the positive modulation of miR-33a-3p, while hMSCs treated with Gefitinib and transfected with the miR-33a-3p inhibitor showed a block of YAP and TAZ modulation by EGFR signaling and consequent downregulation of ERK2 and ERK3 mRNAs expression. These data suggested that miR-33a-3p cooperates with EGFR signaling to regulate Hippo signaling via YAP/TAZ during hMSCs osteoblast commitment (Figure 9).

In conclusion, the current findings provide insightful knowledge regarding the significant role of miRNAs in bone homeostasis and diseases. The understanding of the molecular mechanisms of miR-33a in regulating hMSCs lineage determination might be pivotal to better clarify bone cell differentiation and pathophysiological processes. In the current study, the crosstalk between EGF and YAP signaling mediated by miR-33a-3p during hMSCs osteoblast differentiation was preliminary elucidated. Findings provide novel evidence of miR-33a-5p or 3p as a new molecular target for personalized gene therapies for treating human bone remodeling disorders.

## Figures and Tables

**Table 1:** List of gene primers primers used to study gene expression profiling obtained by Qiagen or custom-made. Their expression was normalized to the β-actin housekeeping gene.

Gene	Qiagen Primers QuantiTect Primer Assay	Catalog Number
*RUNX-2 “Runt-related transcription factor 2”*	Hs_RUNX2_1_SG	QT00020517
*ALPL “Alkaline phosphatase”*	Hs_ALPL_1_SG	QT00012957
*BGLAP “bone gamma-carboxyglutamic acid-containing protein”*	Hs_BGLAP_1_SG	QT00232771
*SPP1 “Secreted Phosphoprotein 1”*	Hs_SPP1_1_SG	QT01008798
**Gene**	**Custom Made Primers Forward**	**Custom Made Primers Reverse**
*SNAIL “Snail family transcriptional repressor 1”*	GCGAGCTGCAGGACTCTAAT	CCCGCAATGGTCCACAAAAC
*SLUG “Snail family transcriptional repressor 2”*	CATGCCTGTCATACCACAAC	GGTGTCAGATGGAGGAGGG
*TWIST “Twist family bHLH transcription factor 1”*	CGGCCAGGTACATCGACTTC	CAGAGGTGTGAGGATGGTGC
*TGF-B “Transforming growth factor beta 1”*	TGGTGGAAACCCACAACGAA	ACACAGAGATCCGCAGTCCT
*YAP “Yes associated protein 1”*	CCTCGTTTTGCCATGAACCAG	GTTCTTGCTGTTTCAGCCGCAG
*TAZ “WW domain containing transcription regulator”*	TGGACCAAGTACATGAACCACC	TCCACAGCCGACTTGTTCTC
*HMGA-2 “High mobility group AT-hook 2”*	GCGCCTCAGAAGAGAGGAC	GTCTTCCCCTGGGTCTCTTAG
*EGFR “Epidermal growth factor receptor”*	CCTATGTGCAGAGGAATTATGATCTTT	CCACTGTGTTGAGGGCAATG
*ERK-2 “Extracellular signal-regulated kinases -2”*	GCGCTACACTAATCTCTCGT	CTGAGGTGCTGTGTCTTCAA
*ERK-3 “Extracellular signal-regulated kinases -3”*	GAATGGCAAATCTGCTCAATT	ACAGTCCTCCCCACCACTCA
**Reference Gene**	**Forward**	**Reverse**
*ACTB “Beta-Actin”*	ATCAAGATCATTGCTCCTCCTGA	CTGCTTGCTGATCCACATCTG

## Supplementary Materials

The following are available online at https://www.mdpi.com/2073-4409/8/12/1495/s1, Figure S1: Densitogram analysis of Slug expression versus α-actin in hMSCs after 24h of transfection with: scramble, mimic-miR-33a-5p and antimiR-33a-5p; Figure S2: Densitogram analysis of Runx2 expression versus α-actin in hMSCs after 24h of transfection with: scramble, mimic-miR-33a-5p and antimiR-33a-5p; Figure S3: hMSCs after 24h of transfection with: scramble, mimic-miR-33a-3p and antimiR-miR-33a-3p were analyzed for the HMGA-2, miR-33a-5p target, expression by qRT-PCR; Figure S4: Densitogram analysis of Slug expression versus a-actin in hMSCs after 24h of transfection with: scramble, mimic-miR-33a-3p and antimiR-33a-3p; Figure S5: Densitogram analysis of Runx2 expression versus α-actin in hMSCs after 24h of transfection with: scramble, mimic-miR-33a-3p and antimiR-33a-3p; Figure S6: Quantitative RT-PCR was performed to evaluate the efficiency of transfection on pre-osteoblast cells (hMSCs maintained in OM medium), after 24h of transfection with: scramble, mimic-miR-33a-5p and antimiRmiR-33a-5p; Figure S7: Study of miR-33a-5p-targets by gain and loss function assays on hMSCs maintained in OM after 24h of transfection with specific mimic and antimir; Figure S8: Densitogram analysis of YAP and TAZ expression versus α-actin in hMSCs after 24h of transfection with: scramble, mimic-miR-33a-5p and antimiR-33a-5p; Figure S9: Densitogram analysis of YAP and TAZ expression versus α-actin in hMSCs after 24h of transfection with scramble, mimic-miR-33a-3p and antimiR-33a-3p; Figure S10: WST-1 assay performed on hMSCs treated with EGF for 24h, 36h and 48h. Data acquired as A540nm and are represented as fold of change compared vs control cells; Figure S11: Densitogram analysis of EGF and ERK1-2 expression versus α-tubulin in hMSCs after 24h, 36h and 48h of EGF treatments; Figure S12: Densitogram analysis of YAP and TAZ expression versus α-tubulin in hMSCs after 24h, 36h and 48h of EGF treatments.
