# Supplementary material for: MiR-33a Controls hMSCS Osteoblast Commitment Modulating the Yap/Taz Expression Through EGFR Signaling Regulation
Source: Cells. 2019 Nov 22;8(12):1495. doi: 10.3390/cells8121495 (PMC6953103; doi:10.3390/cells8121495)
Supplement: Supplementary file 1 [file cells-08-01495-s001.pdf]

Supplementary figures

S1

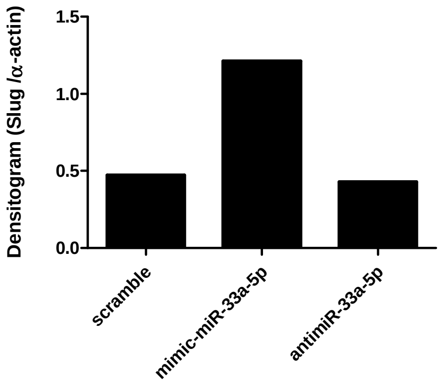

S2

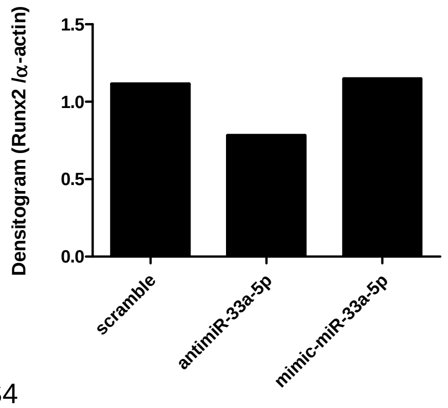

S3

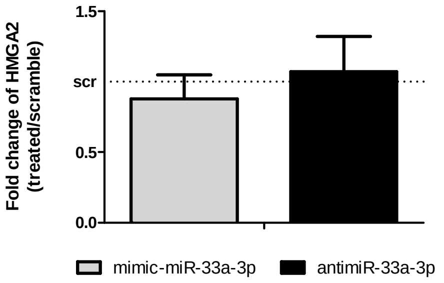

S4

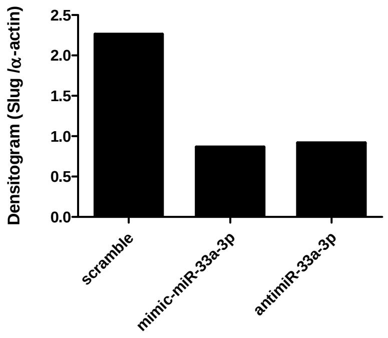

S5

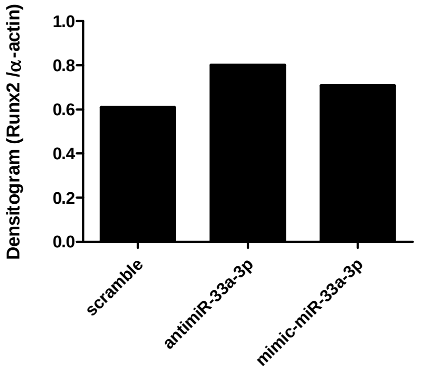

S6

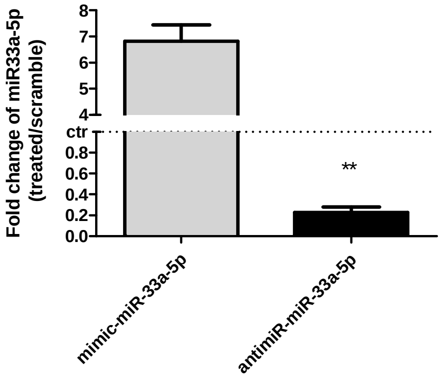

S7

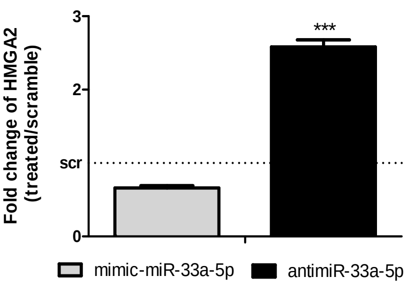

S8

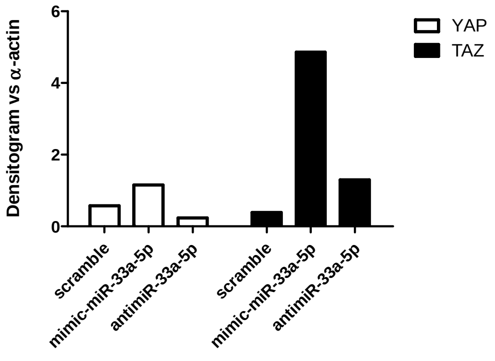

S9

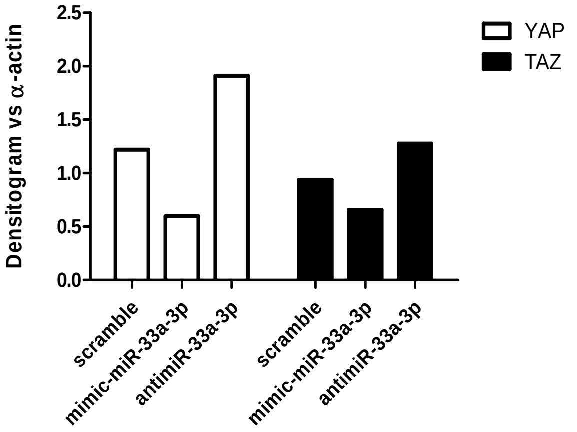

S10

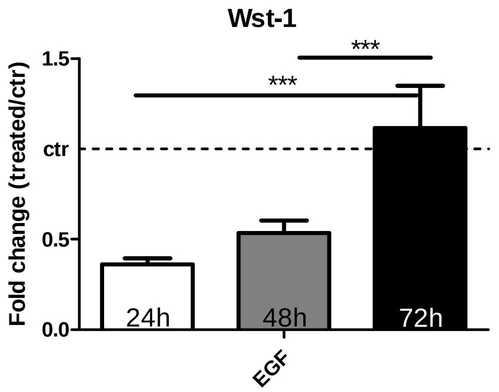

S11

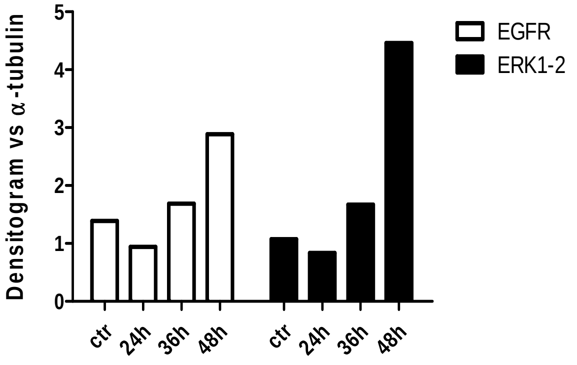

S12

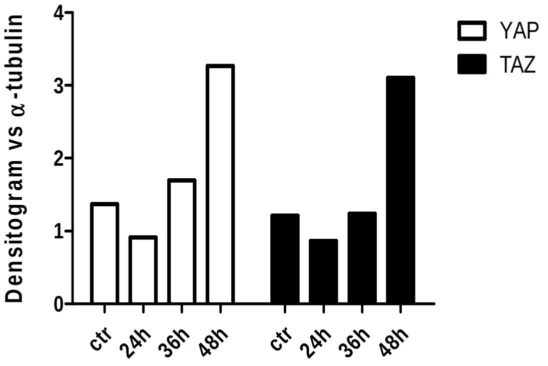

Supplementary Figures Legend

S1: Densitogram analysis of Slug expression versus α-actin in hMSCs after 24h of transfection with: scramble, mimic-miR-33a-5p and antimiR-33a-5p.

S2: Densitogram analysis of Runx2 expression versus α-actin in hMSCs after 24h of transfection with: scramble, mimic-miR-33a-5p and antimiR-33a-5p.

S3: hMSCs after 24h of transfection with: scramble, mimic-miR-33a-3p and antimiR-miR-33a-3p were analyzed for the HMGA-2, miR-33a-5p target, expression by qRT-PCR. Quantitative RT-PCR data are expressed as fold of change (FOI) in gene expression ($2^{-\Delta\Delta Ct}$) occurred in mimic or antimiR vs scramble groups. Student t test: *, p<0.05, **, p<0.005, ***, p<0.0005 between experimental group.

S4: Densitogram analysis of Slug expression versus α-actin in hMSCs after 24h of transfection with: scramble, mimic-miR-33a-3p and antimiR-33a-3p.

S5: Densitogram analysis of Runx2 expression versus α-actin in hMSCs after 24h of transfection with: scramble, mimic-miR-33a-3p and antimiR-33a-3p.

S6: Quantitative RT-PCR was performed to evaluate the efficiency of transfection on pre-osteoblast cells **(**hMSCs maintained in OM medium), after 24h of transfection with: scramble, mimic-miR-33a-5p and antimiR-miR-33a-5p. Quantitative RT-PCR data are expressed as fold of change (FOI) in gene expression ($2^{-\Delta\Delta Ct}$) occurred in mimic or antimiR vs scramble groups. Student t test: *, p<0.05, **, p<0.005, ***, p<0.0005 between experimental group.

S7: Study of miR-33a-5p-targets by gain and loss function assays on hMSCs maintained in OM after 24h of transfection with specific mimic and antimiR. It is analyzed the HMGA-2, miR-33a-5p target, expression by qRT-PCR and data are expressed as fold of change (FOI) in gene expression ($2^{-\Delta\Delta Ct}$) occurred in mimic or antimiR vs scramble groups. Student t test: *, p<0.05, **, p<0.005, ***, p<0.0005 between experimental group.

S8: Densitogram analysis of YAP and TAZ expression versus α-actin in hMSCs after 24h of transfection with: scramble, mimic-miR-33a-5p and antimiR-33a-5p.

S9: Densitogram analysis of YAP and TAZ expression versus α-actin in hMSCs after 24h of transfection with scramble, mimic-miR-33a-3p and antimiR-33a-3p.

S10: WST-1 assay performed on hMSCs treated with EGF for 24h, 36h and 48h. Data acquired as A540nm and are represented as fold of change compared vs control cells.

S11: Densitogram analysis of EGF and ERK1-2 expression versus α-tubulin in hMSCs after 24h, 36h and 48h of EGF treatments.

S12: Densitogram analysis of YAP and TAZ expression versus α-tubulin in hMSCs after 24h, 36h and 48h of EGF treatments.
